# RNA-Seq analysis of interferon inducible p204-mediated network in anti-tumor immunity

**DOI:** 10.1038/s41598-018-24561-2

**Published:** 2018-04-24

**Authors:** Jinlong Jian, Wei Wei, Guowei Yin, Aubryanna Hettinghouse, Chuanju Liu, Yongxiang Shi

**Affiliations:** 1 Shandong Provincial Key Laboratory of Animal Cells and Developmental Biology, School of Life Science, Shandong University, Jinan, 250100 China; 2 Department of Orthopaedic Surgery, New York University School of Medicine, New York, NY 10003 USA; 3 Department of Cell Biology, New York University School of Medicine, New York, NY 10016 USA

## Abstract

p204, a murine member of the interferon-inducible p200 protein family, and its human analogue, IFI16, have been shown to function as tumor suppressors *in vitro*, but the molecular events involved, in particular *in vivo*, remain unclear. Herein we induced the Lewis Lung carcinoma (LLC) murine model of human lung cancer in *p204* null mice (KO) and their control littermates (WT). We compared the transcriptome in spleen from WT and p204 KO mice using a high-throughput RNA-sequencing array. A total 30.02 Gb of clean data were obtained, and overall Q30% was greater than 90.54%. More than 75% of clean data from 12 transcriptome samples were mapped to exons. The results showed that only 11 genes exhibited altered expression in untreated p204 KO mice relative to untreated WT mice, while 393 altered genes were identified in tumor-bearing p204 KO mice when compared with tumor-bearing WT mice. Further differentially expressed gene cluster and gene ontology consortium classification revealed that numerous cytokines and their receptors, chemoattractant molecules, and adhesion molecules were significantly induced in p204 KO mice. This study provides novel insights to the p204 network in anti-tumor immune response and also presents a foundation for future work concerning p204-mediated gene expressions and pathways.

## Introduction

Interferon-inducible p204, encoded by gene *Ifi204*, belongs to the p200 protein family and has been implicated as an important molecule in innate immune response^1,2^. The p200 protein family consists of several interferon-inducible proteins expressed in mice (e.g. p202a, p202b, p203, p204, p205), and humans (e.g. IFI16, myeloid cell nuclear differentiation antigen (MNDA), absent in melanoma 2 (AIM2), and IFIX)^3,4^. Members of the p200 protein family exhibit high structural homology, generally containing an amino-terminus Pyrin domain followed by one or two conserved carboxyl-terminus HIN-200 domains. p200 family members are induced in response to IFN stimulation; the genomic loci of p200 family clusters becomes transcriptionally activated after interferon treatment^5^, and their expression is significantly induced by IFN-γ in myeloid lineage cells^6^, suggesting an important role of p200 proteins in innate immunity. AIM2, a p200 family member, functions as an important inflammasome component that senses potentially pathogenic cytoplasmic DNA, leading to activation of pyroptosome and caspase-1^7,8^. Similarly, IFI16, the human homolog of p204, was discovered to function as a DNA sensor by directly recognizing double-stranded DNA fragments and has been demonstrated to play a vital role in innate immunity against herpes virus infection^9^. Recently we reported that Ifi204 directly binds to TLR4 and it is required to canonical LPS-triggered signaling pathway^10^. Ifi204 KO mice have impaired inflammatory cytokine productions in response to LPS^10^. Therefore Ifi204 plays important function in innate immunity against both virus and bacterial.

Additionally, IFI16 is also required for cell death of human immunodeficiency virus (HIV)-infected CD4+ T cells^11^. p204 and cyclic GMP-AMP synthase (cGAS) cooperate with each other to sense intracellular *Francisella* infection and trigger expression of IFN-γ^12^. Thus, p200 family members play critical roles in viral and bacterial DNA recognition and thereby initiate anti-pathogenic responses. Deregulated p200 family members have reported associations with many auto-immune diseases, including Sjogren’s syndrome (SjS), systemic lupus erythematosus (SLE), systemic sclerosis (SSc) and rheumatoid arthritis (RA)^13,14^. An auto-antibody against IFI16 has been found in Sjogren’s syndrome (SjS)^15^ and scleroderm^16^, and its levels are associated with disease severity.

In addition to its role in regulating innate immunity, p204 is also an important regulator of cell differentiation^17–23^. For instance, the expression of p204 was significantly induced during myoblast fusion, and overexpression of p204 accelerated the fusion of myoblasts to myotubes in both differentiation medium and growth medium^23^. p204 promoted myoblast differentiation, at least in part, by overcoming the inhibition of myoblast differentiation by inhibitor of DNA binding (Id) proteins^24^. p204 directly bound to Id proteins (Id1, Id2, and Id3) through its second HIN-200 domain, and overexpression of p204 resulted in a reduction in the level of Id proteins and the loss of Id-mediated inhibition of MyoD and E47 binding to DNA^24^. p204 is also involved in osteoblast differentiation and overexpression of p204 enhanced BMP-2-induced osteoblast differentiation *in vitro* by acting as a transcriptional coactivator of core-binding factor α 1 (Cbfα1)^22^. Similar results have been observed in adipocytes; knockdown of p204 reduced and overexpression of p204 promoted adipocyte differentiation *in vitro*^25^. The expression of adipocyte markers, such as PPARγ, C/EBP, lipoprotein lipase, and adipsin, was increased by p204 overexpression and decreased by p204 suppression^25^.

p204 also plays an anti-proliferative role in cell cycle regulation through multiple mechanisms. p204 binds to ribosomal RNA-specific UBF1 through HIN-200 domains and inhibits rRNA transcription^26^. Additionally, the two HIN-200 domains of p204 bind retinoblastoma tumor suppressor protein (pRb) and overexpression of p204 in pRB positive cells delayed G0/G1 progression into S phase and impaired E2F-mediated transcriptional activity, while p204 lost its ability to inhibit cell proliferation in pRb null cells, indicating that p204 inhibition of cell proliferation occurs through a pRb-dependent mechanism^27^. Furthermore, p204 directly binds to Ras and may serve as a negative feedback inhibitor of Ras activity^28^. Accordingly, p204 is considered an important tumor-suppressor gene. However, the underlying molecular mechanisms of anti-tumor response mediated by p204 *in vivo* are unclear due to the unavailability of *Ifi204* knockout (KO) mice.

In this study, we present data gathered from the first reported *Ifi204* null mouse line. To investigate the p204 mediated anti-tumor response network, we induced a Lewis Lung carcinoma (LLC) model in both wild type (WT) and p204 KO mice and identified alterations in gene expression using high-throughput RNA sequencing (RNA-seq). We found that p204 is an important regulator of inflammation, chemotaxis, and cell migration in the LLC model. Collectively, this study provides novel insights concerning the IFI204 mediated anti-tumor network.

## Results

### RNA sequencing and transcriptome assembly

**Figure 1:**
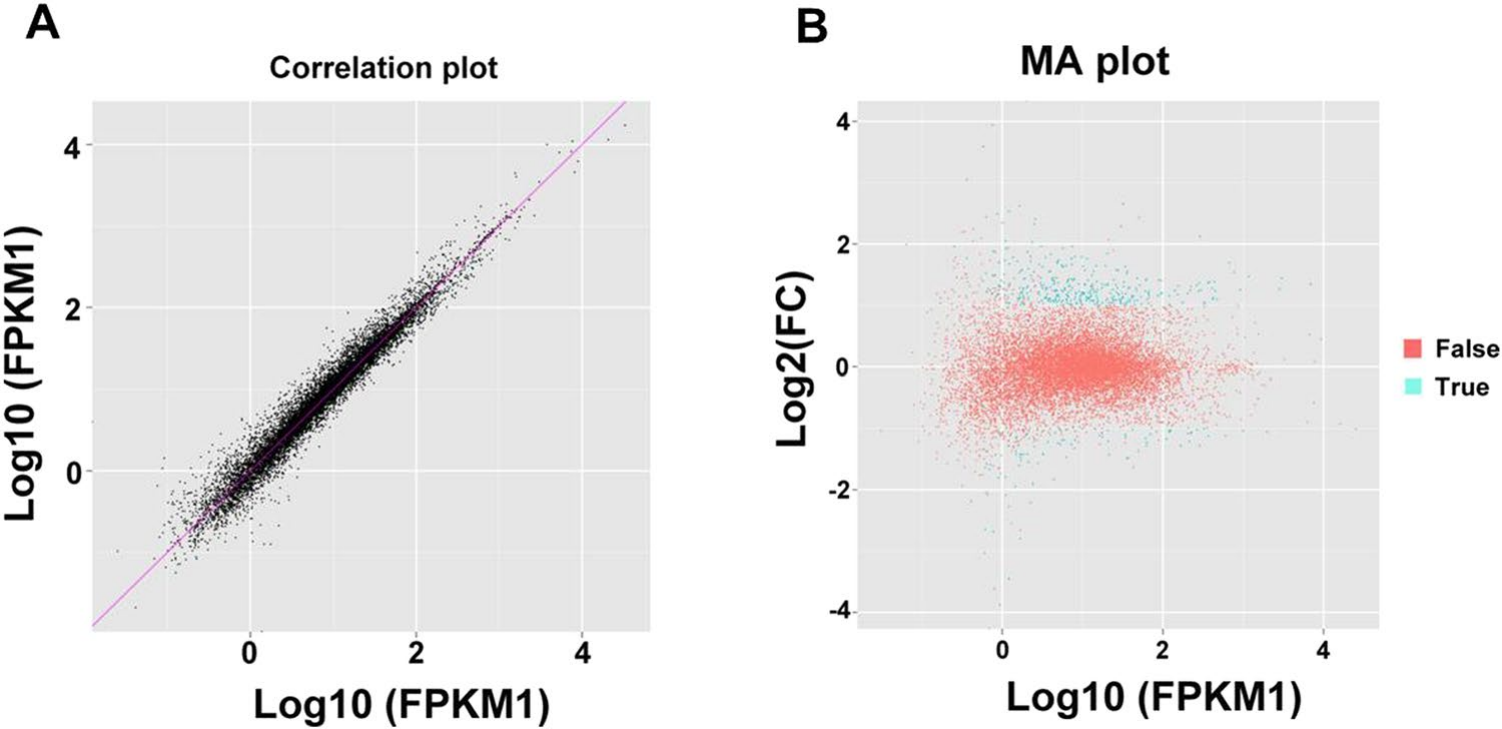
Validation of the RNA-Seq analysis. (**A**) Representative correlation analysis between two samples from the same group. Each group has 3 mice, and transcriptome samples were compared within groups by correlation analysis. (**B**) Representative MA plot to identify genes with significant changes. X-axis (A value) represents Log10 FPKM, and y-axis (M value) represents fold change of each gene between WT and KO. Expression changes larger than 2-fold (log2 > 1) is considered as true hits (green dots), fold changes less than 2-fold (log2 < 1) is considered as (red dots).

To identify the function of p204 in anti-tumor immunity, we injected control group WT and p204 KO mice with PBS and experimental group WT and p204 KO mice with Lewis lung cells (n = 3 per group). 28 days after tumor-inoculation, all mice were sacrificed and spleens were collected for RNA-Seq and transcriptome analysis. Totally, 30.02 Gb of clean data were obtained from 12 mice, and an average of 2.17 Gb clean data were collected from each mouse. The overall Q30 percentage was above 90.54%, more than 90% of readings were mapped to reference genes in all 12 mice, and 75% of readings were located in exons. The average size of mRNA insertion was around 180 bp, and mapping of mRNA fragments to genes generated a smooth curve between 0.1–0.9 relative positions, indicating good quality mRNA and that data retrieved contained enough coverage to represent the overall transcriptome. Gene expression level was represented as FPKM (Fragments per Kilobase of transcript per Million mapped reads) values. The correlation coefficient between every two individual mice was compared, and a correlation coefficient above 0.96 was observed between mice from same group **(**Fig. 1A**)**, indicating that RNA-Seq and analysis is reproducible. MA plot was applied to distinguish true and false differentially expressed genes (DEGs). Changes greater than 2 fold (Log2 > 1) were considered as true hits and changes within two fold were considered as false hits **(**Fig. 1B**)**.

### Functional annotation and characterization of unigenes in control p204 KO mice

Overall, around 4000 DEGs were found in all groups. As shown in Table 1, a total of 11 DEGs, 6 up-regulated and 5 down-regulated, were found in control p204 KO mice when compared with the WT group. The low number of genes evidencing altered expression (11 genes) in control KO mice as compared with control WT mice suggests that p204 may not be required to maintain physiological function under homeostatic conditions. Among the 6 genes up-regulated in control mice, p202, a p200 family member, was significantly induced, and may exhibit compensatory function in the event of p204 deficiency **(**Table 2**)**. However, expression of two other p200 family members, p205 and p204-like protein (or Pyrin domain-containing protein 3, Pydc3) was significantly decreased **(**Table 2**)**. Since these genes sit very close to the *Ifi204* gene on the chromosome, it is speculated that reduced expression of p205 and Pydc3 may be attributable to their impaired transcription resultant of deleting the *Ifi204* gene. The expression of ERDR1, a protein shown to enhance natural killer (NK) cell cytotoxicity^29^, was reduced in p204 KO mice, indicating that p204 may be important for NK cell mediated anti-tumor immunity.

**Table 1:** Summary of DEGs in identified in WT and p204 KO mice.

Groups	All DEG	up-regulated	down-regulated
CtrWT vs CtrKO	11	6	5
CtrWT vs ExpWT	3,292	1,444	1,848
Ctr KO vs ExpKO	3,648	1,528	2,120
ExpWT vs ExpKO	393	303	90

**Table 2:** Genes changed in p204 KO without challenge.

#ID	log2FC	WT vs KO	Swissprot_annotation
**ENSMUSG00000074516**	8.023920047	up	MCG9889 GN = Gm10709
**ENSMUSG00000026535**	1.625195501	up	Interferon-activable protein 202 GN = Ifi202
**ENSMUSG00000038209**	1.526898806	up	Intelectin-1a GN = Itln1
**ENSMUSG00000034656**	1.519550266	up	Voltage-dependent P/Q-type calcium channel subunit alpha-1A GN = Cacna1a
**ENSMUSG00000057897**	1.434858806	up	Calcium/calmodulin-dependent protein kinase type II subunit beta GN = Camk2b
**ENSMUSG00000035202**	1.339684347	up	Probable leucine–tRNA ligase, mitochondrial GN = Lars2
**ENSMUSG00000083929**	−4.058124916	down	Protein Gm10600 GN = Gm10600
**ENSMUSG00000037849**	−3.757042472	down	Protein Gm4955 GN = Gm4955
**ENSMUSG00000026536**	−2.593814803	down	Interferon-activable protein 205-B GN = Ifi205b
**ENSMUSG00000066677**	−1.852480564	down	Pyrin domain-containing protein 3 (interferon-activable protein 204-like) GN = Pydc3
**ENSMUSG00000096768**	−1.462229549	down	Protein Erdr1 GN = Erdr1

### Functional annotation and characterization of unigenes in tumor-bearing IFI204 KO mice

**Figure 2:**
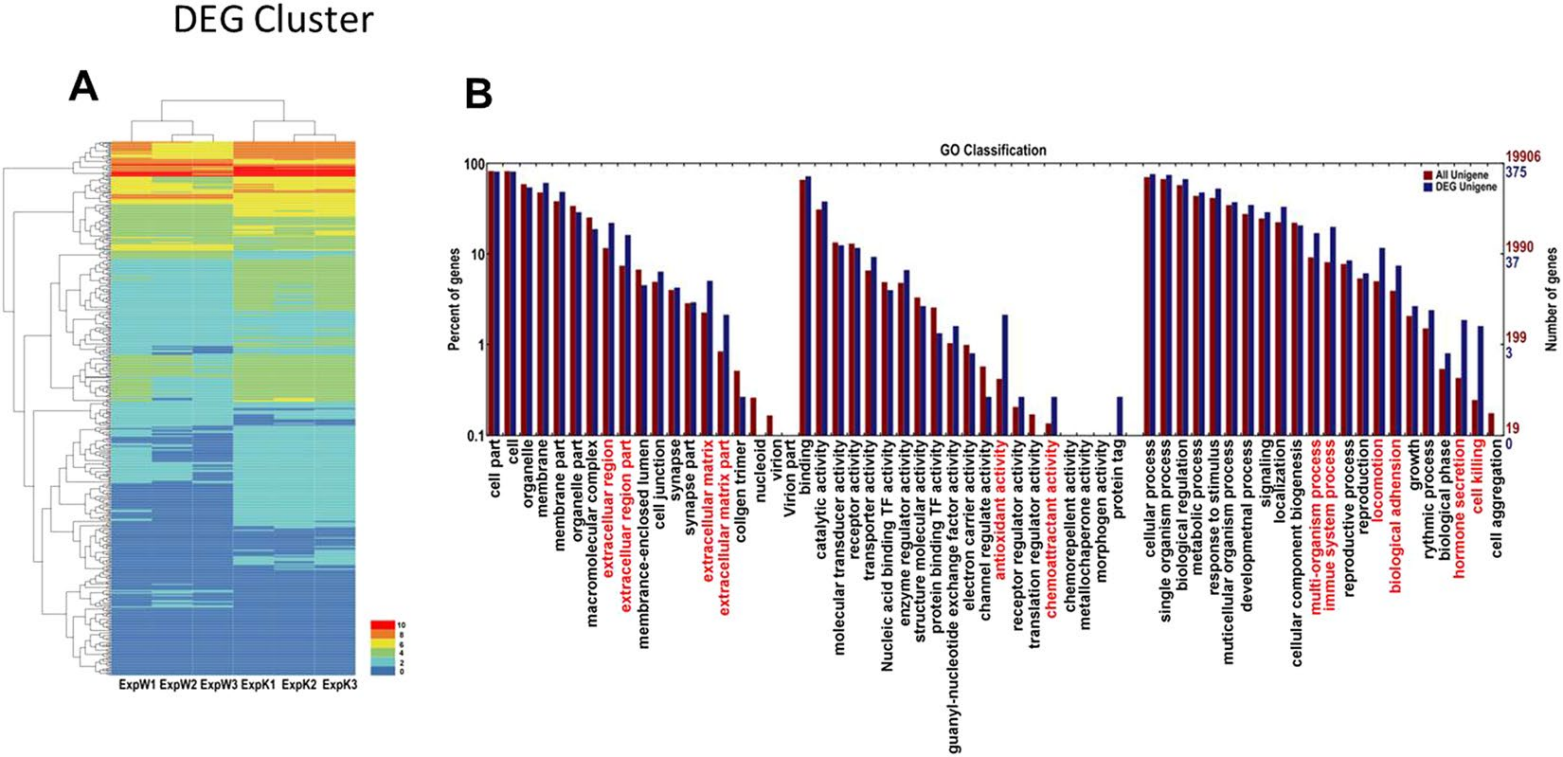
DEG clusters and Gene Ontology categorization for assembled unigenes of the transcriptome. (**A**) DEG clusters in tumor-bearing WT and p204 KO mice. Each column represents each mouse and each row represents each gene. Colors represent the expression level of each gene. (**B**) GP categorization of Unigenes with significant changes in p204. Three mice from each group were compared, and each annotated sequence was assigned at least one GO term in three main categories (biological process, cellular component and molecular function), and 56 subcategories. The x-axis represents the GO term; the y-axis denotes the number of unigenes.

Individually sequenced transcriptomes and DEG expressions from three WT and three p204 KO tumor-bearing mice are indicated in Fig. 2A. The hits from 3 WT mice were compared with those from 3 KO mice, and expression levels of 393 genes were found to be significantly changed. Among these 393 genes, 303 genes were up-regulated and 90 genes were down-regulated **(**Table 1**)**. The heatmap of RNA-Seq shows that expression patterns were very similar across the three samples in each group **(**Fig. 2A**)**. GO functional classes were assigned to the cluster DEGs with putative functions. These genes were sorted into major functional categories that exhibit significant overall changes in p204 KO mice; categories include genes involved in extracellular matrix, anti-oxidant activity, chemoattractant activity, multi-organism process, immune process, adhesion, and cell killing activities **(**Fig. 2B**)**. Selected genes with significant change are listed in Table 3.

**Table 3:** Selected gene changes in tumor-bearing p204 KO mice.

#ID	log2FC	WT vs KO	Swissprot_annotation
**IFI Family members**			
ENSMUSG00000037849	−3.87635	down	Pyrin domain-containing protein 3 GN = Pydc3
ENSMUSG00000026536	−3.45103	down	Interferon-activable protein 205-B GN = Ifi205b
ENSMUSG00000034459	−1.41452	down	Interferon-induced protein with tetratricopeptide repeats 1 GN = Ifit1
ENSMUSG00000026535	3.58602	up	Interferon-activable protein 202 GN = Ifi202
**Cytokine and its receptors**			
ENSMUSG00000015966	3.046982	up	Interleukin-17 receptor B (Precursor) GN = Il17rb
ENSMUSG00000044103	1.758106	up	Interleukin-36 gamma (Precursor) GN = Il36g
ENSMUSG00000026073	1.552664	up	Interleukin-1 receptor type 2, soluble form (Precursor) GN = Il1r2
ENSMUSG00000062157	1.334625	up	Interferon lambda receptor 1 (Precursor) GN = Ifnlr1
ENSMUSG00000003206	1.149416	up	Interleukin-27 subunit beta (Precursor) GN = Ebi3
ENSMUSG00000026981	1.126317	up	Interleukin-1 receptor antagonist protein (Precursor) GN = Il1rn
ENSMUSG00000071714	1.083263	up	Interleukin-3 receptor class 2 subunit beta (Precursor) GN = Csf2rb2
ENSMUSG00000026070	1.009005	up	Interleukin-18 receptor 1 (Precursor) GN = Il18r1
**Extracellular matrix**			
ENSMUSG00000056481	−1.070622	down	Endosialin (Precursor) GN = Cd248
ENSMUSG00000029304	2.492212	up	Osteopontin (Precursor) GN = Spp1
**Anti-oxidant activity**			
ENSMUSG00000021792	−1.213679	down	Redox-regulatory protein FAM213A GN = Fam213a
ENSMUSG00000056054	1.446364	up	Protein S100-A8 GN = S100a8
ENSMUSG00000031722	1.366310	up	Haptoglobin beta chain (Precursor) GN = Hp
ENSMUSG00000056071	1.344669	up	Protein S100-A9 GN = S100a9
**Chemoattractant**			
ENSMUSG00000048480	2.347394	up	C-X-C chemokine receptor type 1 GN = Cxcr1
ENSMUSG00000026180	1.375646	up	C-X-C chemokine receptor type 2 GN = Cxcr2
ENSMUSG00000025804	1.330659	up	C-C chemokine receptor type 1 GN = Ccr1
**Multi-organism process**			
ENSMUSG00000006464	−1.29234	down	Bardet-Biedl syndrome 1 GN = Bbs1
ENSMUSG00000034226	1.28859	up	Rho-related GTP-binding protein RhoV
**Leukocytes adhesion**			
ENSMUSG00000026580	1.632646	up	P-selectin (Precursor) GN = Selp
ENSMUSG00000074272	1.570366	up	Carcinoembryonic antigen-related cell adhesion molecule 1 GN = Ceacam1
ENSMUSG00000040950	1.546964	up	C-type lectin domain family 10 member A GN = Clec10a
ENSMUSG00000030786	1.402912	up	Integrin alpha-M (Precursor) GN = Itgam
ENSMUSG00000034028	1.399784	up	CD226 antigen (Precursor) GN = Cd226
ENSMUSG00000029915	1.367813	up	C-type lectin domain family 5 member A GN = Clec5a
ENSMUSG00000030142	1.243278	up	C-type lectin domain family 4 member E GN = Clec4e
ENSMUSG00000030342	1.193822	up	CD9 antigen GN = Cd9
ENSMUSG00000000157	1.169239	up	Integrin beta-2-like protein (Precursor)
ENSMUSG00000020689	1.163936	up	Integrin beta-3 (Precursor) GN = Itgb3
ENSMUSG00000027111	1.157775	up	Integrin alpha-6 light chain (Precursor) GN = Itga6
ENSMUSG00000030144	1.137084	up	C-type lectin domain family 4 member D GN = Clec4d
ENSMUSG00000053063	1.026039	up	C-type lectin domain family 12 member A GN = Clec12a
**Cell killing activities**			
ENSMUSG00000014543	2.403747	up	Killer cell lectin-like receptor 5 GN = Klra5
ENSMUSG00000015950	1.315022	up	Neutrophil cytosol factor 1 GN = Ncf1
ENSMUSG00000057729	1.098486	up	Myeloblastin (Precursor) GN = Prtn3

**Figure 3:**
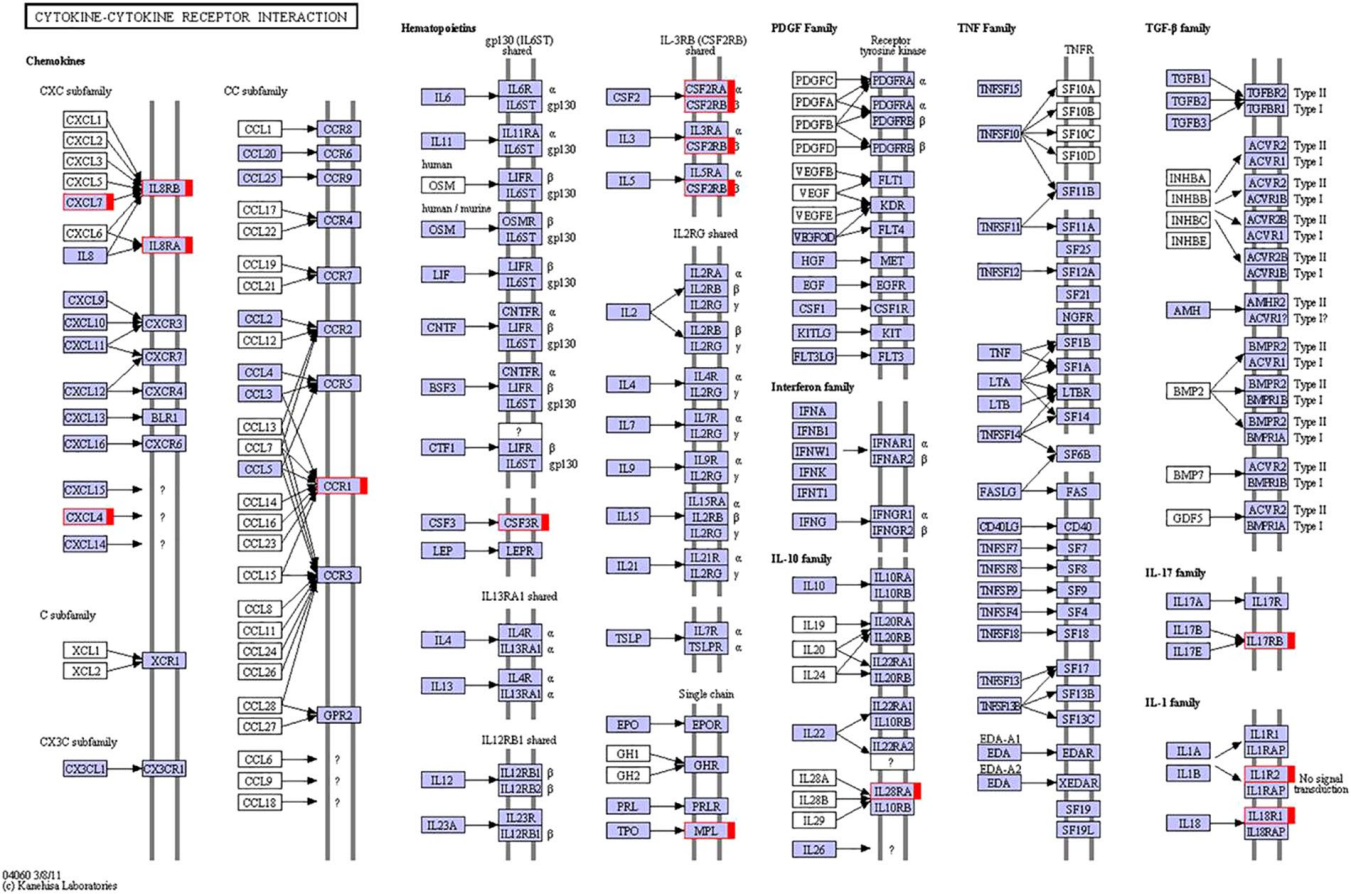
Changes of cytokine-related DEGs and pathways in tumor-bearing p204 KO mice. Cytokine-related DEGs and pathway were analyzed by KEGG database (http://www.kegg.jp/kegg/kegg1.html) as descripted previously^31,32^. Unigenes up-regulated in p204 KO mice are indicated by red boxes.

Consistent with the control group, p200 family members were also significantly affected in tumor-bearing p204 KO mice; p202 was dramatically induced, while p205b, Pydc3, and IfIT1 were reduced **(**Table 3**)**. The consistency of changes in expression for p200 family members in experimental and control groups strengthens our data and indicates the validity of experimental procedure and bioinformatics analysis. In addition to p200 family members, cytokines and their receptors, including IL-17RB, IL-36γ, and type 2 IL-1R, IL-27, IL-18R and IL-3R (selective list in Table 3), were also significantly affected. Among cytokine receptors, IL17RB is most significantly induced with an approximate 8-fold increase. IL-17RB is the receptor of IL-17B and IL17E (IL-25) and their interaction is critical to T helper 2 cell (Th2) immune response^30^. Robust induction of ILI7RB suggests that p204 may be an important molecule in the regulation of Th2 immune response. IL-1 family members, IL-1R2 and IL-18R and IL-36γ, were all significantly induced in tumor-bearing p204 KO mice (Table 3, Fig. 3), suggesting that p204 is also an important regulator of inflammation. Additionally, several chemokine receptors and leukocyte adhesion molecules, including CXCR1, CXCR2, CCR1, and integrin molecules and c-type lectins were up-regulated in p204 KO mice **(**Table 3**)**. The increased expression of chemoattractant molecules and leukocyte adhesion molecules may promote tumor growth and metastasis in p204 KO mice. DEGs of those cytokines and receptors were further analyzed by KEGG (Kyoto Encyclopedia of Genes and Genomes) pathway annotation^31,32^. As shown in Fig. 3, chemokines from both the CXC subfamily and CC subfamily, IL-3RB receptors, IL-17 family and IL-1 family members were most induced in p204 KO mice. This data suggests that there may be aggravated inflammation in tumor-bearing p204 KO mice than in WT mice.

**Figure 4:**
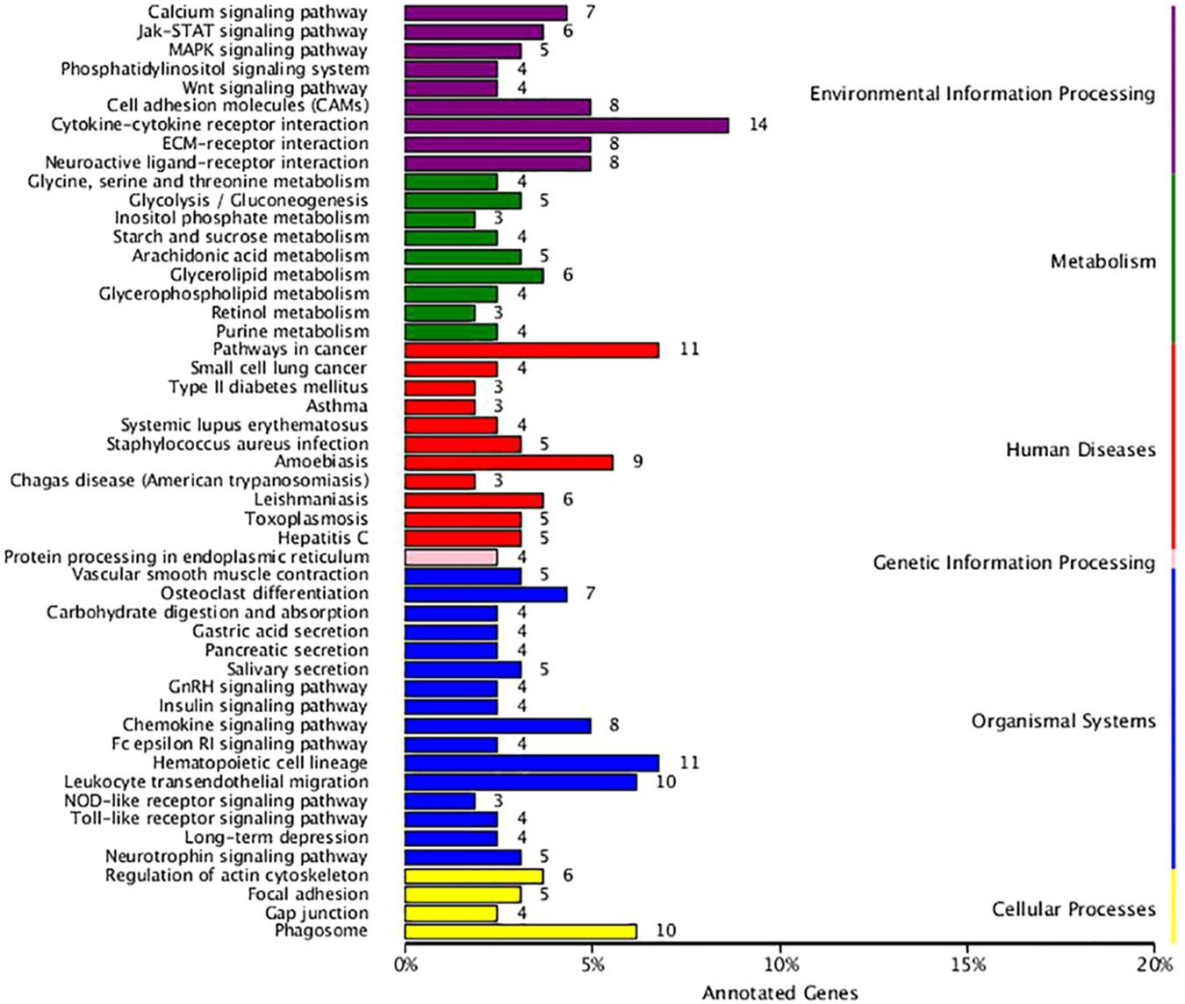
Distribution of DEGs in representative pathways as compared between tumor-bearing WT and p204 KO mice. KEGG enrichment analysis (http://www.kegg.jp/kegg/kegg1.html) was performed to identify pathways mediated by p204.

To understand p204 mediated pathways, DEGs were analyzed against the KEGG database for pathway enrichment^31,32^. Between each of the treatment groups and the control, a total of 150 pathways were enriched; the greatest proportion of DEGs are involved in cytokine-receptor interaction, pathways in cancer, hemapoeitc cell linage, leukocyte transendothelial migration, and phagosome pathway **(**Fig. 4**)**. The networks mediated by p204 were also analyzed by topGO, enrichment analysis for Gene Ontology. As shown in Table 4, most DEGs affected by deficiency of p204 have functions integral to ATP binding, protein binding, zinc ion binding, protein homodimerization, identical protein binding, protein domain specific binding, protein kinase binding, and protein complex binding. This information suggests that p204 may be a critical signaling molecule for multiple cellular events, such as protein dimerization and protein complex formation.

**Table 4:** DEG topGO Functional Analysis.

GO. ID	Term	Annotated	Significant	Expected	KS
GO:0005524	ATP binding	1635	360	247.33	<1e-30
GO:0005515	protein binding	8506	1546	1286.7	<1e-30
GO:0008270	zinc ion binding	1221	227	184.7	2.0e-20
GO:0042803	protein homodimerization activity	876	161	132.51	3.1e-16
GO:0042802	identical protein binding	1326	249	200.58	4.1e-16
GO:0019904	protein domain specific binding	838	159	126.76	5.3e-15
GO:0019901	protein kinase binding	574	128	86.83	3.2e-13
GO:0032403	protein complex binding	602	107	91.06	3.3e-13

Note: GO. ID: gene ontology ID; Term: GO function; Annotated; number of all the genes annotated in each GO ID have specific function; significant: number of DEG annotated in each GO ID; Expected: expected number of DEG in each GO.

## Discussion

Anti-viral and anti-tumor immunity are key tasks of immune system that are essential for protection from virus-mediated infectious disease^33^ and the development of cancer^34^. Interferon-inducible p200 family members have been found to be critical molecules for initiating anti-viral immune response^1,7–9,11^. Human IFI16 and its murine homolog, p204, have been specifically identified as viral DNA sensors that recognize double stranded DNA from herpes simplex virus (HSV)^9^ and HIV^11^. Interestingly, IFI16 was also reported to bind to single stranded DNA^35^, at an even higher affinity than it does with double strand DNA. Deficiency of IFI16 leads to defective response to viral infection. AIM2, another p200 family member, binds to IFI16 and plays central role in triggering inflammasome pathway response to viral infection^7,8^. It is proposed that HIN-200 domains function as a docking site that mediates polymerization among p200 family members, as well as with other proteins, allowing formation of large protein assemblies which act as the molecular machinery for initiation of innate immune response^36^. It is very clear that the p200 family is essential to innate immunity. However, the function of p200 family members in anti-tumor immunity has remained unexplored. In this study, we take advantage of the first established *Ifi204* null mouse line and induction of a mouse model of human lung cancer to analyze the p204-mediated network in anti-tumor immunity using high throughput RNA-Seq.

Interestingly, we report that gene expression profiles of p204 KO and WT mice treated with PBS revealed differential expression of only 11 genes, suggesting that p204 function may not be required to maintain normal physiological function. This discovery is consistent with findings that p204 KO mice are generally normal under physiological conditions. However, significant upregulation from low basal levels of p204 was observed following stimulation by interferon and poly I:C^37^, viral infection^38^, and bacterial components^37,39^, suggesting that p204 is likely to be an important molecule for immune response in certain pathological conditions, such as viral infection. Indeed, significant DEGs were observed in tumor-bearing p204 KO mice compared with tumor-bearing WT mice. Of a total 393 genes evidencing altered expression levels in mice induced under the LLC model, 303 were up-regulated and 90 were down-regulated in p204 KO mice relative to WT mice. Chemokines and their receptors, as well as leukocyte adhesion molecules were most significantly affected gene product categories. Increased levels of chemotaxis and cell migration are associated with cancer metastasis^40,41^, thus, p204 may inhibit both functions to suppress cancer growth and metastasis. These findings are also in line with the previous *in vitro* data demonstrating p204 as a tumor-suppressor^27^. Overexpression of p204 has been shown to inhibit cell growth, delay cell transition from G1 to S phase, and impair DNA synthesis through binding to pRb, a tumor suppressor protein, and mutated p204 lacking the pRb binding motif loses its effect on cell-cycle regulation^27^. Many cytokines and their receptors were also significantly induced in p204 KO mice subjected to LLC (Fig. 3). It is well known that increased levels of inflammation are expected to promote cancer cell proliferation and metastasis in many types of cancer^42–44^, upregulation of cytokines and cytokine receptors in LLC-induced, p204 KO mice suggests that p204 may limit inflammation in the LLC model, which may be another mechanism by which p204 suppresses tumor progression.

p204 and its human analogue, IFI16, are key signal molecules that mediate viral infection, and both molecules contain conserved Pyrin and HIN domains^2^. Both Pyrin and HIN domains are critical for protein-protein interaction, polymerization and formation of inflammasome^36,45,46^. In topGO analysis, we also found that p204 is critical for multiple protein binding events; including ATP binding, zinc binding, protein homodimerization, identical protein binding, kinase binding, and protein complex binding **(**Table 4**)**. These findings indicate that p204 not only mediates inflammasome formation, but may also be involved in other cellular events such asregulation of zinc finger transcription factor activity, controlling intracellular signaling through binding to protein kinases, and mediating protein dimerization. These results are supported by previous *in vitro* findings that p204 bound to and regulated numerous transcription factors, including UBF1, MyoD, GATA4 and Cbfa1^20–22,24,26^. In addition, these clues warrant additional research to better describe these potential functions of p204.

In summary, we utilized high-throughput RNA-Seq to investigate the p204-mediated gene expression profile and signaling network under the Lewis lung carcinoma model using the first reported p204 KO mice. The results reveal that p204 significantly affects chemotaxis and cell migration, as well as cytokine expression. Our findings support previous reports of p204′s role in infection and indicate that p204 may also play an important role in anti-tumor immunity. This study provides an exciting expositional framework for future studies concerning the role of p204, and its human counterpart IFI16, in anti-tumor immune response. This study also provides new evidence concerning additional functionality of p204 in cellular signaling cascades, inflammation, and hormone secretion. Overall, these findings shed light on potential directions for future biological studies of p204.

## Methods

### Ethics statement

All the experimental procedures were performed in accordance with Guide for the Care and Use of Laboratory Animals^47^ and were approved by the ethics committee in Shandong University.

### Generation of *Ifi204*^−/−^ Mice

The characterization of *Ifi204* KO mice were reported previously^10^. Briefly, we used mouse strain 129 to generate loxP-floxed p204 mice in which exon2 and exon5 of the *Ifi204* gene were flanked by loxP sequences. The floxed p204 mice were then crossed with Sox2-Cre mice (which directly express Cre in epiblast at E6.5) to generate *p204*+/− mice. For the purpose of genetic background consistency, *IfI204*^+/−^ mice were used as parental mice to produce mice of *IfI204*^−/−^ (KO) and *IfI204*^+/+^ (WT) genotypes. *IfI204*^−/−^ (KO) mice were backcrossed with C57BL/6 for 10 generations before used for *in vitro* and *in vivo* experiments.

### *Ifi204* KO mice and Lewis Lung model

WT and *Ifi204* KO mice were kept in the animal facility at Shandong University, and The Lewis lung carcinoma model was induced as reported previously^48,49^. Briefly 8-week-old male WT and *Ifi204* KO mice were injected with 2 × 10^6^ Lewis Lung cells subcutaneously in right axilla, and control mice were injected with PBS. Induction of the mouse tumor model was considered successful when protrusions at the cell injection site could be palpated a week after injection. Mice were sacrificed at 28 days after tumor implantation. Spleens were collected for RNA-Seq analysis.

### RNA extraction, library construction, and RNA-Seq

RNA was extracted from the samples according to the instruction manual for the TRIzol reagent (Invitrogen, Carlsbad, CA). RNA concentration and purity was measured using the NanoDrop 2000 Spectrophotometer (Thermo Fisher Scientific, Wilmington, DE). RNA integrity was assessed using the RNA Nano 6000 Assay Kit of the Agilent Bioanalyzer 2100 system System (Agilent Technologies, CA, USA).

High-quality RNA was sent to Biomarker Technologies Corporation (Beijing, China) for cDNA library construction and sequencing. mRNA was purified by magnetic oligo (dT) beads. RNA sequencing libraries were generated using the NEBNext® Ultra RNA Library Prep Kit for Illumina (New England Biolabs, Ipswich, MA, U.S.A.) with multiplexing primers, according to the manufacturer’s protocol. The cDNA library was constructed with average inserts of 200 bp (150~250 bp) following a non-stranded library preparation protocol. The cDNA was purified using an AMPure XP Beads (Beckman Coulter, Inc.). The short cDNA fragments were subjected to end repair followed by adapter ligation. Suitable fragments were selected by Agencourt AMPure XP beads (Beckman Coulter, Inc.) and enriched by PCR amplification. Sequencing was performed via a paired-end 125 cycle rapid run on the Illumina HiSeq. 2500.

### Transcriptome analysis using reference genome-based reads mapping

Low quality reads, such as adaptor only, unknown nucleotides >5%, or Q20 <20% (representative percentage of sequences with sequencing error rates <1%), were removed by Perl Script programming. The clean reads that were filtered from the raw reads were mapped to mouse (C57BL/6 strain) reference genome (GRCm38) using Tophat2 software^50^. Gene expression levels were estimated using FPKM (Fragments Per Kilobase of exon per Million fragments mapped) values using Cufflinks software^51^.

### Identification of differential gene expression

DESeq.^52^ and Q-value were employed to evaluate differential gene expression between groups. After that, gene abundance differences between those samples were calculated based on the ratio of FPKM values. Multiple testing correction via the false discovery rate (FDR) was used to identify the threshold of the p-value across multiple tests in order to assess the significance of the differences. Here, only genes with an absolute value of log2 ratio ≥2 and FDR significance score <0.05 were used for subsequent analysis.

### Sequence Annotation

Genes were compared against various protein databases, including the National Center for Biotechnology Information (NCBI) non-redundant protein (Nr) database and the Swiss-Prot database, by the basic local alignment search tool (BLASTX) with a cut-off E-value of 10-5. Genes were retrieved based on the best BLAST hit (highest score) along with their protein functional annotation.

To annotate the gene with gene ontology terms, the Nr database BLAST results were imported into the Blast2GO program^53^. This analysis mapped all of the annotated genes to GO terms in the database and counted the number of genes associated with each term. Perl script was then used to plot GO functional classification for the unigenes with a GO term hit in order to view the distribution of gene functions. The obtained annotation was enriched and refined using TopGo (R package). The gene sequences were also aligned to the Clusters of Orthologous Group (COG) database to predict and classify functions^54^. Kyoto Encyclopedia of Genes and Genome (KEGG) pathways were assigned to the assembled sequences by Perl Script programming.
